# Predicting Structural Details of the Sodium Channel Pore Basing on Animal Toxin Studies

**DOI:** 10.3389/fphar.2018.00880

**Published:** 2018-08-07

**Authors:** Denis B. Tikhonov, Boris S. Zhorov

**Affiliations:** ^1^Sechenov Institute of Evolutionary Physiology and Biochemistry, Russian Academy of Sciences, Saint Petersburg, Russia; ^2^Department of Biochemistry and Biomedical Sciences, McMaster University, Hamilton, ON, Canada

**Keywords:** conotoxins, homology modeling, ligand docking, local anesthetics, tetrodotoxin

## Abstract

Eukaryotic voltage-gated sodium channels play key roles in physiology and are targets for many toxins and medically important drugs. Physiology, pharmacology, and general architecture of the channels has long been the subject of intensive research in academia and industry. In particular, animal toxins such as tetrodotoxin, saxitoxin, and conotoxins have been used as molecular probes of the channel structure. More recently, X-ray structures of potassium and prokaryotic sodium channels allowed elaborating models of the toxin-channel complexes that integrated data from biophysical, electrophysiological, and mutational studies. Atomic level cryo-EM structures of eukaryotic sodium channels, which became available in 2017, show that the selectivity filter structure and other important features of the pore domain have been correctly predicted. This validates further employments of toxins and other small molecules as sensitive probes of fine structural details of ion channels.

## Introduction

**FIGURE 1:**
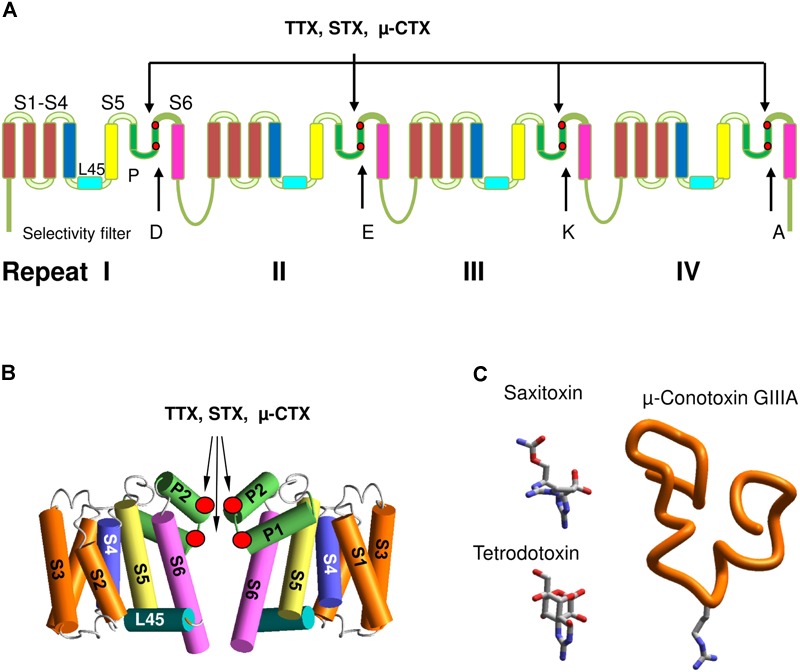
Eukaryotic sodium channels and the outer pore blockers. **(A)** α-Subunit transmembrane topology. Positions of the selectivity filter DEKA residues and the outer carboxylates, which interact with TTX, STX and μ-CTX are marked by red dots. **(B)** General 3D model of sodium channels. Positions of toxin-interacting residues are shown by red circles. **(C)** 3D structures of the outer pore blocking toxins. Sown is Arg13 in GIIIA, which is critical for the channel block.

Voltage-gated sodium channels belong to the superfamily of voltage-gated ion channels, which also include calcium, potassium, glutamate-gated, and other channels. Eukaryotic VGSCs comprise α and β subunits. The large α-subunit, which folds from a single polypeptide chain of four homologous repeats, contains a pore domain and four VSDs domains. Each repeat comprises six transmembrane helical segments (S1–S6) connected by extra- and intracellular loops. Segments S1–S4 form VSDs. Segments S5 (the outer helices), S6 (the inner helices), and extracellular membrane reentering P-loops between S5 and S6 contribute to the pore domain (**Figure 1A**). The P-loops contain membrane-descending (P1) and membrane-ascending (P2) helices with residues between P1 and P2 contributing to the selectivity filter. In eukaryotic VGSCs, the selectivity filter DEKA ring, which contains D, E, K, and A residues, borders the extracellularly exposed outer pore and the inner pore that is exposed to the cytoplasm in the open channel (**Figure 1B**). The activation gate, which is composed of the cytoplasmic parts of S6s, forms a tight ion-impermeable bundle in the closed state. Upon membrane depolarization the S4 helices, which contain positively charged residues, shift in the extracellular direction, thus inducing movements of the S4–S5 linker helices and finally the activation gate opening. In the open state, S6s diverge to form a wide inner vestibule.

Voltage-gated sodium channels play key roles in physiology of excitable cells and pathology of nerves and muscle (Hille, 2001). The sodium ions influx to the cell further depolarizes the membrane, thus generating the rising phase of the action potential. Within few milliseconds after their opening, the channels rapidly inactivate in the process known as fast inactivation. Following membrane repolarization, VGSCs recover from inactivation and the activation gate closes (Catterall, 2012). After prolonged membrane depolarization (seconds to minutes), VGSCs enter into slow-inactivated states, the process important for regulating membrane excitability, action potential patterns, and spike frequency adaptation (Vilin and Ruben, 2001; Ulbricht, 2005). Due to their widespread expression and critical functions in electrically excitable cells, VGSCs are targets for deadly toxins, which are synthesized by poisonous organisms as the attack or/and defense weapons (Catterall, 1980). Multiple diseases are associated with mutations in sodium channels (Catterall, 2014; Huang et al., 2017; Sanders et al., 2018). All these hallmarks have made VGSCs the subject of intensive studies in academia and industry. One of goals of these studies is development of various drugs including local anesthetics, antiarrhythmics, anticonvulsants, and antidepressants. The drug design and development requires high-resolution 3D structures of the channels. Thirteen years after the breakthrough publication of the KcsA potassium channel structure (Doyle et al., 1998), structure of the prokaryotic sodium channel, NavAb became available (Payandeh et al., 2011). Recently, NMR has been used to explore interactions of toxins with VSDs of the Nav1.4 channel (Mannikko et al., 2018). Cryo-EM structures of the cockroach sodium channel NavPaS (Shen et al., 2017) and electric eel Nav1.4 channel (Yan et al., 2017) provide currently the most comprehensive structures.

Despite the impressive progress in structural studies, high-resolution 3D structures are still unavailable for mammalian VGSCs. Rational drug design also requires knowledge of the channel structures in different functional states (open, closed, fast-inactivated, and slow-inactivated). Furthermore, structures of the channel mutants underlying channelopathies and multiple drug-channel complexes in different channel states are unlikely to become available in the foreseeable future. This makes theoretical methods, which help integrate results of experimental studies and propose testable predictions, an important component of structural studies. However, computational approaches have serious limitations. Thus, docking of a ligand into a known 3D structure of an ion channel does not necessarily yield an unambiguous ligand-channel model. Problems that are difficult to resolve with standard computational protocols include multiplicity of possible ligand–protein complexes, unknown ionization state of residues, and missing or misinterpreted ions or water molecules. Among other factors that are difficult to take into account are membrane voltage, auxiliary subunits, and entropy component of free energy. As a result, computational studies produce sometimes controversial models of ligand-bound channels (Zimin et al., 2010; Marzian et al., 2013) despite each model is consistent with certain set of experimental data. Therefore, comparison of theoretical models with later published experimental structures is important.

Here, we review application of theoretical approaches for understanding details of the pore organization in VGSCs. We describe how integration of various experimental data, including data on toxin action allowed predicting fine details of the pore structure, including the selectivity filter geometry, which in view of the NavPaS and Nav1.4 structures proved correct.

## Molecular Probes of the Sodium Channel Structure

Many ideas of molecular organization of VGSCs were elucidated from the analysis of the channel interactions with small molecules and peptides, whose structures are available or predictable. Even before the amino acid sequences of ion channels were published (Numa and Noda, 1986), important conclusions about the molecular organization of ion channels had been made. For example, the dimensions of the selectivity filter where proposed to match those of the largest permeant organic cations (Hille, 1971). Structure-function studies of ligands reveled the binding site organization (see Hille, 2001). Particularly, analysis of local anesthetics’ action supported the conclusion on two access pathways for some drugs into the channel pore: a hydrophilic pathway from inside the cell through the open activation gate and a hydrophobic pathway from the membrane (Hille, 1977). With advent of the molecular biology era, the transmembrane topology of VGSCs was deduced from analysis of hydrophobicity (Noda and Numa, 1987).

Mutational studies helped to determine roles of individual residues in toxin binding (Terlau et al., 1991) that in turn allowed to propose a 3D scheme of toxin binding site (Lipkind and Fozzard, 1994). The era of X-ray crystallography of ion channels opened a possibility to build homology models of sodium channels and move from schematic representation to more realistic 3D homology models. The key roles in these indirect studies were played by highly specific toxins, which belong to various categories and target different sites. The channel activators (agonists) include batrachotoxin, veratridine, aconitine and grayanotoxins, which according to recent data bind inside the pore, stabilize the open state, but do not prevent the ion permeation (Tikhonov and Zhorov, 2005b; Wang et al., 2006). The mechanism of batrachotoxin action (Du et al., 2011) resembles that of a surgical stent in the blood vessel. Another group of toxins includes peptides produced by scorpions (Wang et al., 2011; Zhang J.Z. et al., 2011; Zhang J.Z. et al., 2012), spiders (Bosmans and Swartz, 2010; Minassian et al., 2014) and sea anemones (Xiao et al., 2014). These toxins bind between the pore domain and VSDs and modify the channel activation and inactivation by various toxin-specific mechanisms. Mutagenesis and modeling studies of scorpion alpha- and beta-toxins suggested that VSD-IV and VSD-II are positioned, respectively, close to the pore domain helices in repeats I and III (Leipold et al., 2006; Cohen et al., 2007; Wang et al., 2011; Chugunov et al., 2013). TTX, STX, and μ-CTXs were critically important for structural studies of the outer pore (**Figure 1C**).

## Tetrodotoxin and Saxitoxin

Action of these small semi-rigid toxins on VGSCs is reviewed by many authors (e.g., Hille, 2001; Moczydlowski, 2013; Thottumkara et al., 2014). Briefly, by the early 1960s it had became clear that the “puffer fish poison” and “clam poison” contain small molecules that in nanomolar concentrations reversibly block initiation and propagation of the action potential. The blocking effects of TTX and STX on electrical excitability were systematically studied (e.g., Dettbarn et al., 1960; Narahashi et al., 1960; Evans, 1964). The chemical structures of TTX and STX were later determined (Woodward and Gougoutas, 1964; Schantz et al., 1975). TTX and STX were proposed to block the ion permeation by physically occluding the sodium channel pore (Kao and Nishiyama, 1965). Bertil Hille integrated available experimental data in a schematic model where TTX and STX blocked the sodium channel vestibule (Hille, 1975a,b). In this model, the toxin guanidinium group fit into the selectivity filter lined by six oxygen atoms that form an anionic ring of 3–5 Å in diameter. The model predicted at least five hydrogen bonds between the toxin and the channel. Subsequent site-directed mutagenesis of the channel protein revealed at least 10 residues in the four channel repeats whose substitutions reduce the TTX and STX affinity by more than twofold.

Intensive mutational studies revealed that TTX and STX binding sites include selectivity-filter residues in the DEKA ring and outer carboxylates three to four positions downstream from the DEKA residues (**Figure 1A**). In combination with structure-activity studies of toxin derivatives, the mutational analysis helped to determine pairwise contacts between the channel residues and chemical groups of TTX and STX (**Figures 1A,B**). In particular, the DEKA ring and outer carboxylates were found to be critical for the action of TTX and STX (Terlau et al., 1991). Later TTX and STX were shown to similarly interact with the selectivity filter residues, but differently with the outer carboxylates (Penzotti et al., 1998). Tomaselli and coauthors mapped TTX-sensing residues in P-loops by cysteine scanning (Yamagishi et al., 2001). Further studies revealed important details of TTX and STX action in different sodium channels (Choudhary et al., 2003; Bricelj et al., 2005; Jost et al., 2008; Walker et al., 2012). Substitutions of a tyrosine residue C-terminal to the repeat I aspartate with unnatural amino acids demonstrated a strong contribution of cation–pi interactions in TTX binding (Santarelli et al., 2007).

## Conotoxins

More than 700 cone snail species from at least 16 genetically distinct superfamilies produce conotoxins, diverse peptides often synthesized with post-translational modifications. Conotoxins are important research tools and potential therapeutic substances. Currently, only ∼0.1% of conotoxins are characterized pharmacologically. Conotoxins have many different molecular targets, including various ion channels (Lewis et al., 2012). Four classes of VGSC-targeting conotoxins (μ, μO, δ, and L) have been isolated from cone snail venoms (Cruz et al., 1985; Fainzilber et al., 1994; McIntosh et al., 1995; Fiedler et al., 2008). These conotoxins have different mechanisms of action. Two classes (μ and μO) inhibit and two classes (δ and L) activate VGSCs (Green and Olivera, 2016).

Best characterized are μ-CTXs isolated from the venom of piscivorous *Conus* species (Green et al., 2014). The folding of μ-CTXs is stabilized by three disulfide bridges arranged in a type III framework (CC-C-C-CC). The μ-CTXs binding site overlaps with that of TTX and STX (Fozzard and Lipkind, 2010), but since μ-CTXs are larger than TTX and STX, they exhibit greater specificity for VGSC isoforms. Most of μ-CTXs are positively charged molecules, which are electrostatically attracted to the acidic residues in the VGSC outer vestibule. In several μ-CTXs, a single basic residue, e.g., Arg13 in GIIIA (**Figure 1C**), is critical for the ion current block (Chang et al., 1998; Hui et al., 2002). Other basic residues in μ-CTXs control their orientation and binding to the channel.

Mutational analysis of the channel and μ-CTXs revealed their specific pairwise contacts (Chang et al., 1998; Xue et al., 2003) and the clockwise arrangement of the four repeats at the extracellular view (Dudley et al., 2000; Li et al., 2001; Choudhary et al., 2007). Furthermore, the mutant cycle determined the free energy of interactions for certain toxin-channel contacts (Li et al., 2001; Choudhary et al., 2007). Binding sites of different μ-CTXs essentially overlap despite the fact that patterns of residue-residue interactions are not identical. All the outer carboxylates, which interact with permeant ions (Khan et al., 2002), are key components of the binding sites for TTX, STX, and μ-CTXs. Interestingly, whereas TTX and STX completely block the current, residual currents are observed in the channel complexes with some native and mutant μ-CTXs (Hui et al., 2002; McArthur et al., 2011a; Wilson et al., 2011). Recently, the LRET spectroscopy was used to estimate distances between μ-CTX bound in the central pore and VSDs in their resting and activates states (Kubota et al., 2017).

## Sodium Channel Models Based on X-Ray Structures of Potassium Channels

Lipkind and Fozzard proposed a pioneering structural model of the TTX and STX receptor (Lipkind and Fozzard, 1994) 4 years before the first crystal structure of an ion channel (KcsA potassium channel) was published (Doyle et al., 1998). The model employed the data that action of TTX and STX is dramatically reduced by substitutions of the selectivity-filter aspartate and glutamate, and the outer carboxylates in repeats I, II, and IV (Terlau et al., 1991). In this model, anti-parallel hairpin-like segments from the four P-loops form a funnel-like toxin binding region. The TTX guanidinium group binds to the selectivity filter and a hydroxyl group forms an H-bond with the outer carboxylate in repeat II. TTX and STX reside in the cavity between the four hairpins and interact with other residues that, according to experimental data, are expected to contribute to the toxins binding site. At the extracellular view of this model, repeats I, II, III, and IV are arranged clockwise. This fundamental prediction has been proven with the mutant cycle analysis of the pairwise residue interactions of μ-CTX GIIIA and the channel (Dudley et al., 2000).

**FIGURE 2:**
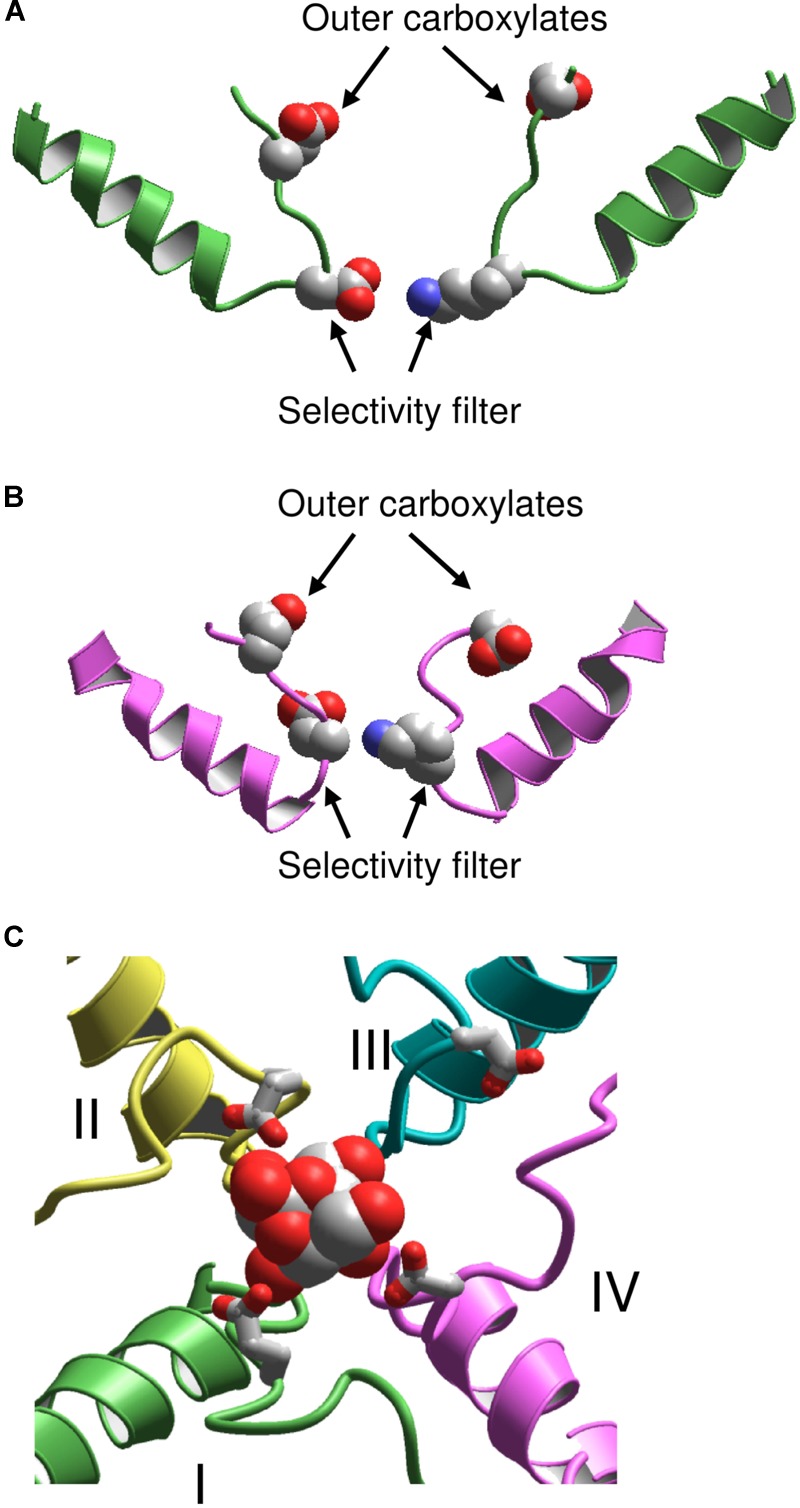
KcsA-based models of sodium channels. **(A)** Model (Lipkind and Fozzard, 2000) in which the P helices are shifted from the pore axis as compared with KcsA and the DEKA selectivity filter is located at the P-loop turn. **(B)** Model (Tikhonov and Zhorov, 2005a) in which the P-helices are disposed as in KcsA, and the DEKA ring is located in the middle of the ascending limbs. **(C)** Extracellular view at the TTX-bound model (Tikhonov and Zhorov, 2012). Repeats I, II, III, and IV are green, yellow, cyan, and magenta, respectively. The outer carboxylates are shown as sticks.

Following publication of the KcsA X-ray structure, Lipkind and Fozzard elaborated a homology model of the Nav1.4 channel and docked TTX and STX using experimental data on toxin-channel interactions (Lipkind and Fozzard, 2000). However, the outer pore of KcsA appeared too narrow to accommodate the bulky semirigid toxins. To resolve the problem, the selectivity-filter DEKA residues were positioned at the border between the central cavity and the outer pore, at the C-ends of P-helices (**Figure 2A**). The P-helices along with the outer pore-lining ascending limbs were shifted farther from the pore axis as compared to KcsA. To visualize known pairwise contacts between large μ-CTXs and the channel, the KcsA-based model of Nav1.4 was further modified by shifting the P-helices even farther from the pore axis and increasing their slope relative to the pore axis (Choudhary et al., 2007).

A stronger 3D similarity between potassium and sodium channels was assumed in the alternative KcsA-based model of Nav1.4 with TTX and STX (Tikhonov and Zhorov, 2005a). In this model, the DEKA residues are placed in the middle of the ascending limbs, and the P-helices remained in positions, which are seen in KcsA (**Figure 2B**). Intensive Monte Carlo energy minimizations yielded the toxin-channel complexes where TTX and STX fit snugly into the outer pore (**Figure 2C**). Despite the different placements of the DEKA residues and different positions of P-helices, the mutual disposition of the DEKA ring and the ring of outer carboxylates are similar in both models, which were built to integrate the same set of experimental data on toxin-channel contacts.

## Hydrophobic Access Pathway for Local Anesthetics

Despite the fact that binding site of local anesthetics in the central cavity is far from the binding sites for TTX and μ-CTXs in the outer vestibule, the toxins helped to resolve a paradox of the closed channel block by local anesthetics. That some drugs can block VGSCs by reaching their binding sites via a hydrophobic pathway was initially proposed basing on the analysis of structure-function relations of local anesthetics and related compounds (Hille, 1977). Permanently charged quaternary compounds block closed cardiac VGSCs, but not neuronal and skeletal-muscle isoforms (Alpert et al., 1989; Qu et al., 1995). Mutational data suggested critical roles of some residues in the III/IV repeat interface. Localization of this pathway between transmembrane helices IIIS6 and IVS6 and IIIP was predicted in a computational study (Tikhonov and Zhorov, 2005a). More recent computations (Boiteux et al., 2014), which used the X-ray structure of bacterial sodium channel NavAb (Payandeh et al., 2011) agree with this prediction.

**FIGURE 3:**
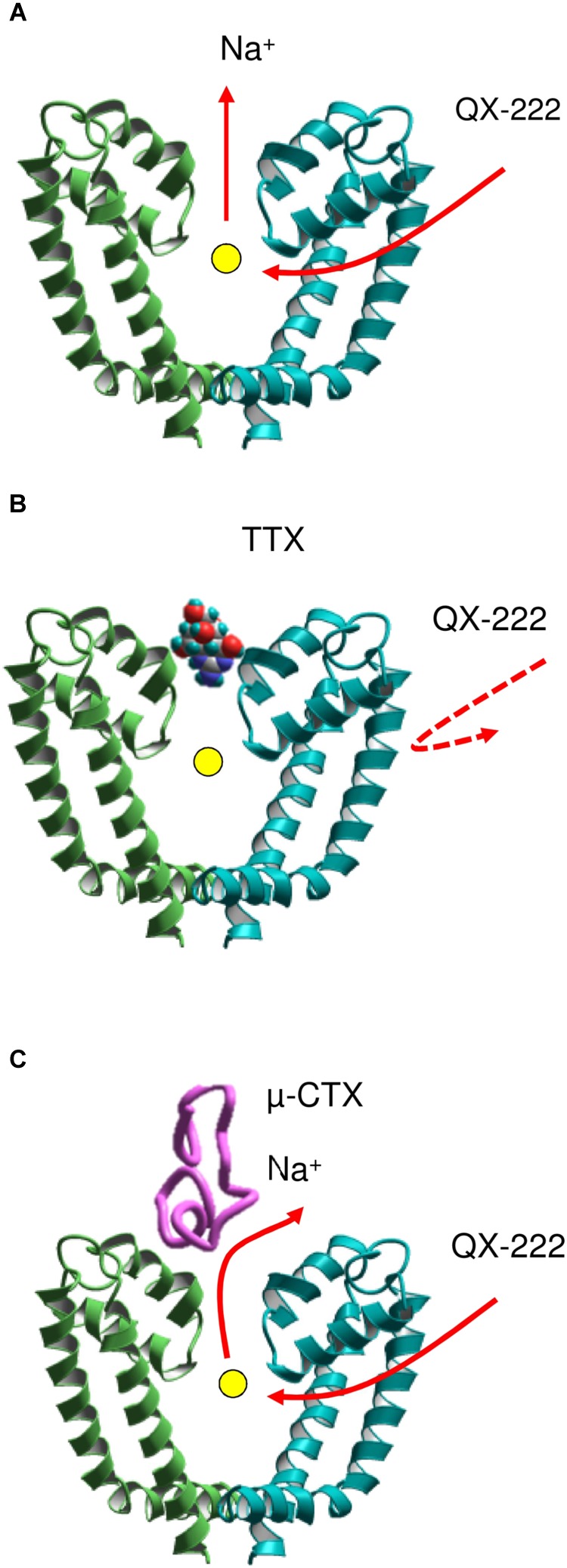
A scheme of coupled movement of a sodium ion and ligand QX-222 in the closed cardiac sodium channel (Bruhova et al., 2008). **(A)** The permanently charged QX-222 reaches the inner pore via III/IV repeat interface and displaces the resident ion that leaves the central cavity through the selectivity filter. **(B)** When the outer pore is blocked by TTX, the sodium ion is trapped in the central cavity and prevents binding of QX-222. **(C)** A μ-CTX mutant does not completely block the sodium current. The sodium ion can escape through the incompletely sealed outer pore and vacate the cation-attractive central cavity for binding of QX-222.

However, it was unclear why TTX (Qu et al., 1995), but not a GIIIA mutant (Sunami et al., 2001) prevents the closed channel block by a permanently charged local anesthetic-like ligand, QX-314. At first sight, this observation suggests that the access pathway to the closed channel involves the outer pore. A solution of this paradox was proposed in a model (**Figure 3**) that combines two features: (i) the access pathway for local anesthetics into the closed channel through the membrane-exposed III//IV interface and (ii) a sodium ion residing in the central cavity of the TTX-bound channel (Bruhova et al., 2008). The electronegative focus of P1 helices would be attractive for cationic local anesthetics unless it is occupied by another cation. Thus, the permanently charged drug targeting the cavity should displace from it the resident sodium ion (**Figure 3A**). In the TTX-bound closed channel, the sodium ion residing in the central cavity lacks any way to leave it: the activation gate is closed, the hydrophobic pathway is prohibitive for the hydrated ion, and the outer-pore route is blocked by TTX. Therefore, quaternary compounds cannot reach the binding site due to repulsion from the ion (**Figure 3B**). When the GIIIA mutant R13N binds to the channel, some residual current is observed (Sunami et al., 2001). Therefore, when the local anesthetic displaces the sodium ion, it can escape the central cavity to the extracellular space by moving between polar residues of the channel and the GIIIA mutant (**Figure 3C**).

## Progress Inspired by X-Ray Structures of Prokaryotic Sodium Channels

In the X-ray structures of bacterial sodium channels (Payandeh et al., 2011; Zhang X. et al., 2012) the selectivity filter glutamates are in the middle of the ascending limbs and positions of P1-helices are close to those in potassium channels. These experimental structures supported the assumption on close 3D similarity of membrane-descending P-helices in sodium and potassium channels (Tikhonov and Zhorov, 2005a). Furthermore, the X-ray structures demonstrated wide fenestrations between subunits that could provide a spacious access pathway for some ligands from the membrane into the inner pore. Thus, the predicted localization of the hydrophobic access pathway for some drugs (Tikhonov and Zhorov, 2005a; Bruhova et al., 2008) has been also confirmed.

An important model-based prediction was presence of a sodium ion at the cation-attractive focus of P1 helices (Tikhonov et al., 2006). In proposed models this ion lacks direct contacts with the channel residues, and binds to various ligands including batrachotoxin (Tikhonov and Zhorov, 2005b; Du et al., 2011), local anesthetics and anticonvulsants (Tikhonov et al., 2006; Bruhova et al., 2008; Tikhonov and Zhorov, 2017b; Buyan et al., 2018). Now a completely hydrated sodium ion Na_III_ is seen in the focus of four backbone carbonyls at the border of the outer pore and the inner pore, close to the focus of P1 helices (Naylor et al., 2016). Such location makes the ion available for direct contacts with ligands targeting the central cavity.

**FIGURE 4:**
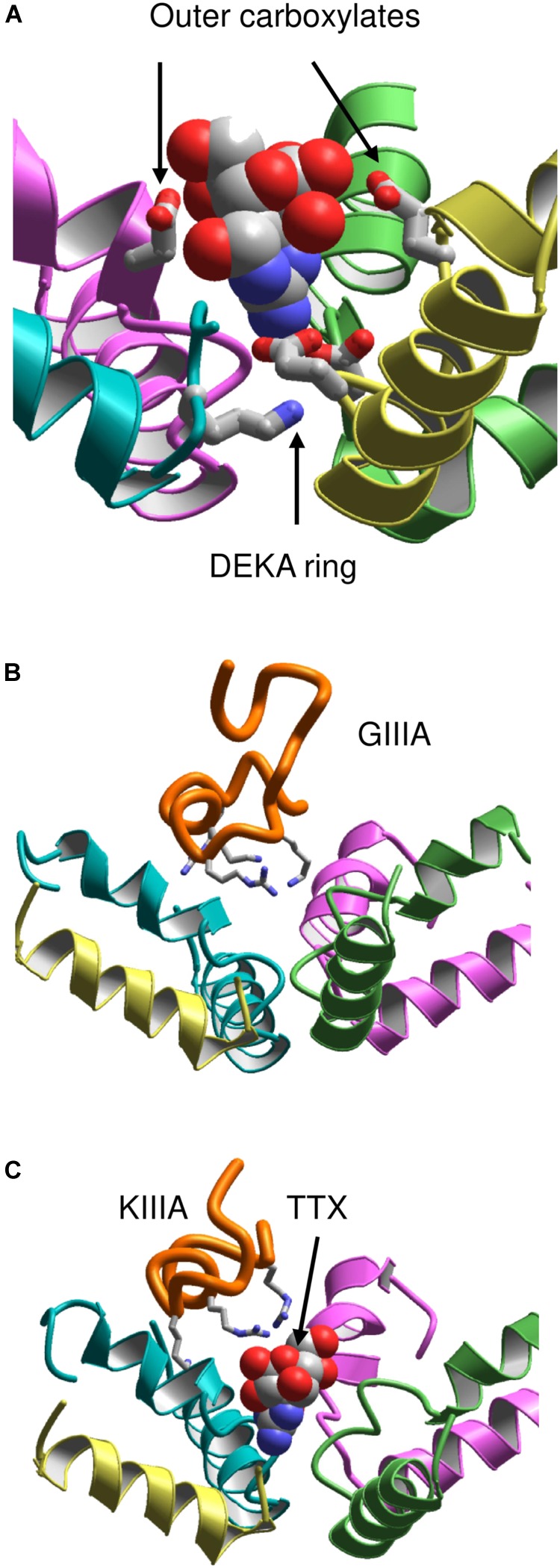
Toxins in the NavAb-based models of Nav1.4. **(A)** TTX fits between the DEKA ring and the outer carboxylates (Tikhonov and Zhorov, 2012). Helix P2 in repeat III is removed for clarity. **(B)** μ-CTX GIIIA (orange) binds in the wide outer vestibule and positively charged toxin residues (shown as sticks) form salt-bridges with the channel outer carboxylates (Korkosh et al., 2014). **(C)** A small toxin KIIIA bound in the outer pore leaves space for binding of TTX (Korkosh et al., 2014).

It should be noted that models based on the X-ray structures of potassium channels failed to predict that the C-terminal halves of P-loops contain membrane-ascending P2 helices, which make the outer pore wide enough to accommodate large toxins. Therefore, the NavAb structure motivated new theoretical studies aimed to rationalize binding of toxins to the outer pore. However, a NavAb-based model of Nav1.4 built using the straightforward sequence alignment of P-loops (Tikhonov and Zhorov, 2012) failed to explain mutational data on TTX interactions with the outer carboxylates, which faced away the pore axis and did not interact with the toxin. To resolve the problem, deletions near the DEKA residues were introduced in the Nav1.4 sequence aligned with NavAb (Tikhonov and Zhorov, 2012). The model built with this alignment re-oriented the outer carboxylates so that their contacts with TTX (**Figure 4A**) are consistent with experimental data summarized by Lipkind and Fozzard.

This Nav1.4 model was further employed to dock μCTXs GIIIA, PIIIA, and KIIIA (Korkosh et al., 2014). The distance constraints between GIIIA and the channel residues, which represent pairwise interactions revealed by the mutant cycle analysis (Choudhary et al., 2007), facilitated the toxin docking. These constraints have been satisfied without deforming the toxin and the channel backbones (**Figure 4B**). Correlation between the computed and experimental energies of specific interactions (Choudhary et al., 2007) further validated the model. The voltage-dependence of action of PIIIA and its mutants helped determine the depths of individual residues in the membrane electric field (McArthur et al., 2011b). The model of PIIIA-Nav1.4 complex (Korkosh et al., 2014) is in a good agreement with these data.

Some native and mutant conotoxins incompletely block the current (Hui et al., 2002; McArthur et al., 2011a; Wilson et al., 2011). For the first time the model (Korkosh et al., 2014) proposed the following rationale for this interesting phenomenon: TTX fits the narrow part of the outer pore and completely plugs it, whereas large μCTXs bind above the outer pore and cover, but do not plug it. The ion permeation is blocked by the μCTX charged residues that form salt bridges with the outer carboxylates. If the channel-bound toxin lacks some of the basic residues, at least one carboxylate would not form a salt bridge with the toxin and provide a transient binding site for permeating ions. The latter would pass through the outer pore of toxin-bound channel, although the ion current would be smaller than that in toxin-free channels. Intriguingly, TTX and KIIIA can simultaneously bind to the sodium channel (Zhang et al., 2009; Van Der Haegen et al., 2011; Wilson et al., 2011; Khoo et al., 2012; Stevens et al., 2012). In agreement with these data, the model simultaneously accommodates both TTX and KIIIA (Korkosh et al., 2014) (**Figure 4C**).

## Homology Models vs. Cryo-EM Structures of Eukaryotic Sodium Channels

**FIGURE 5:**
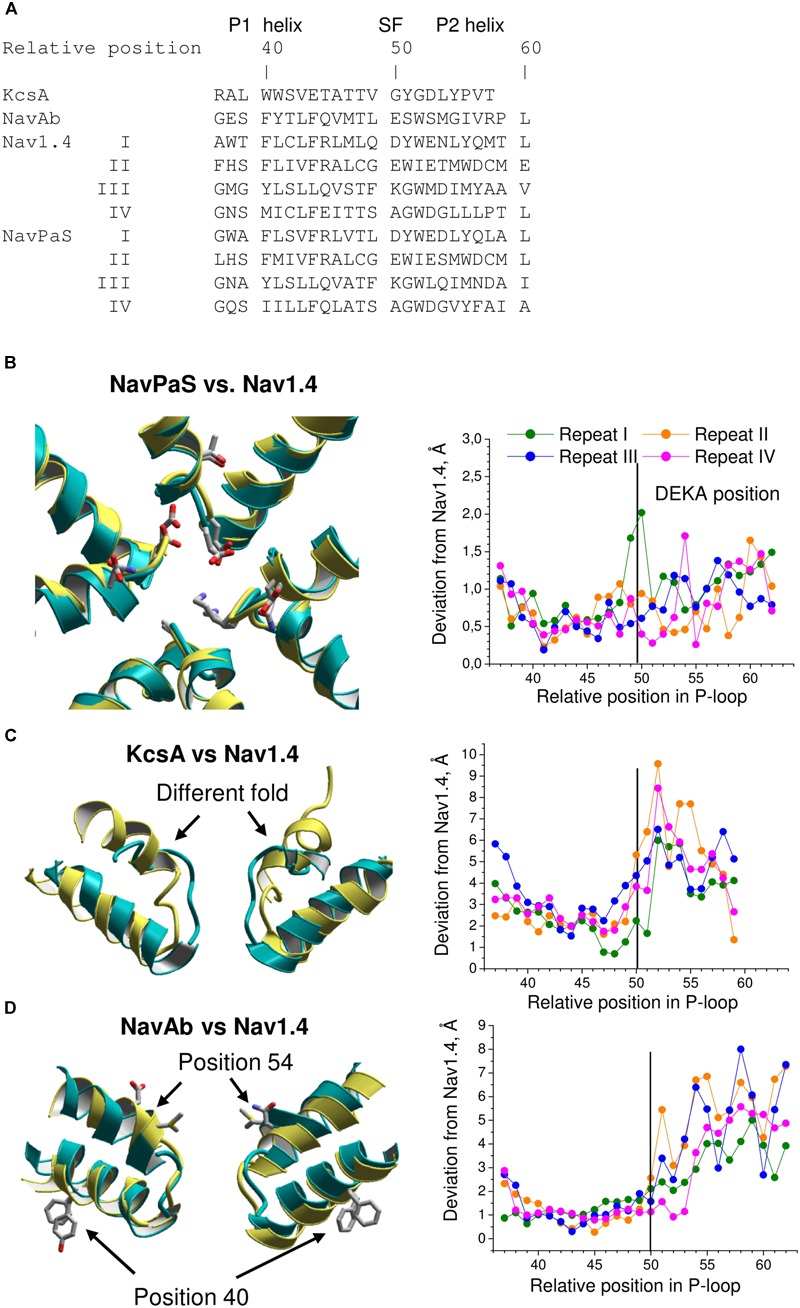
Comparison of P-loops in Nav1.4, NavPaS, KcsA, NavAb. **(A)** Straightforward sequence alignment of P-loops. In the NavPaS and Nav1.4 sequences the repeat numbers are indicated by roman numerals. The top row shows relative positions of residues in the aligned sequences; position 50 is assigned to the selectivity-filter DEKA residues. Comparison of NavPaS **(B)**, KcsA **(C)**, and NavAb **(D)** structures with Nav1.4. The structures are 3D aligned by minimizing RMS deviations of alpha carbons in the P1 helices from matching atoms in Nav1.4 according to the sequence alignment shown in **(A)**. Left panels show 3D aligned structures with Nav1.4 in yellow. Right panels are plots of deviations of alpha carbons from matching atoms in Nav1.4. **(B)** NavPaS is similar to Nav1.4 and most deviations are less than 1.5 Å. The selectivity filter residues and the outer carboxylates are shown as sticks in the superimposed structures. **(C)** KcsA significantly deviates from Nav1.4 in the outer pore region where sodium and potassium channels have different folding. **(D)** NavAb and Nav1.4 have similar P1-helices, but large deviations are seen in the P2 helices where amino acids in matching positions (according to the straightforward sequence alignment) have different orientations in the 3D-aligned structures.

The above homology models have been built using certain assumptions and experimental data whose interpretations are ambiguous. Furthermore, precision of computational models of complex transmembrane proteins is limited. Given these limitations, correctness of the homology models was questionable. Now the cryo-EM structures of VGSCs allow judging the accuracy of the modeling predictions. To obtain the accuracy criteria, we first compared 3D aligned structures of Nav1.4 with NavPaS, KcsA, and NavAb. The 3D alignment was obtained by minimizing RMS deviations of alpha carbon atoms in residues, which according to the straightforward sequence alignment are in matching positions of the P1 helices (**Figure 5A**). The P1 helices are chosen for the 3D alignment because they are the most structurally conserved segments of P-loop channels (Tikhonov and Zhorov, 2017a).

The 3D aligned NavPaS and Nav1.4 are very similar and most deviations between matching atoms do not exceed 1.5 Å (**Figure 5B**). The largest deviations correspond to the selectivity filter aspartate in repeat I and the outer carboxylate in repeat IV. The deviations generally increase at the N-end of P1 and C-end of P2 where the helices are connected to flexible loops. Functional groups at the ends of long flexible sidechains are expected to deviate more than alpha carbons. Indeed, distances between terminal groups of the residues, which contribute to the TTX binding site, vary from 0.7 to 2.7 Å (**Table 1**). We further compared Nav1.4 with KcsA and NavAb whose X-ray structures were used as templates to model eukaryotic sodium channels. The P1 helices in potassium and sodium channels have different slope relative to the pore axis and the deviation distances between alpha carbons of matching residues vary from 1 to 3 Å. The distances sharply increase to 5–9 Å at the outer-pore region, which folds differently in sodium and potassium channels (**Figure 5C**).

**Table 1:** Deviations (Å) of central atoms of TTX-binding functional groups in homology models and NavPaS from matching atoms in the Nav1.4 cryo-EM structure.

Residue	Model/structure			
	Lipkind and Fozzard, 2000	Tikhonov and Zhorov, 2005a	Tikhonov and Zhorov, 2012	NavPaS
Y^1p51^	3.7	6.1	2.2	0.5
E^1p53^	10.3	8.1	2.4	0.7
E^2p53^	5.4	4.6	3.9	2.7
D^3p54^	4.6	6.6	6.1	2.4
D^4p53^	8.8	9.5	2.3	0.8
D^1p50^	4.2	3.0	4.4	2.0
E^2p50^	2.9	2.7	1.9	0.8
K^3p50^	1.1	1.6	1.5	2.0
A^4p50^	1.7	2.1	1.4	0.4

**FIGURE 6:**
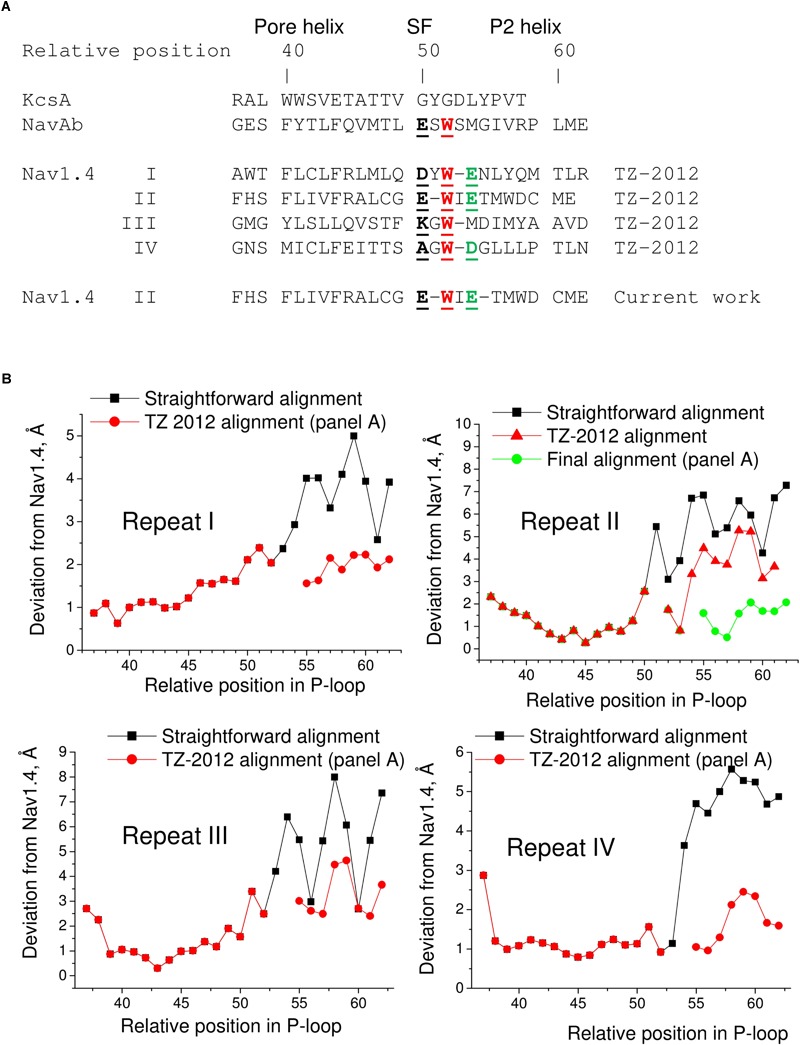
Adjusted sequence alignment decreases deviations of matching atoms in 3D aligned structure of NavAb. **(A)** Adjusted sequence alignment (TZ-2012) of P-loops in KcsA, NavAb, and Nav1.4 (Tikhonov and Zhorov, 2012). Highlighted are residues in the DEKA locus, conserved tryptophans that stabilize folding of the P-loops, and the outer carboxylates. In the adjusted, but not the straightforward (**Figure 5A**) sequence alignment the conserved tryptophans are in the matching positions. The second deletion in repeat II of Nav1.4 is introduced in the current work to minimize deviations between matching alpha carbons in the P2 helices of NavAb and Nav1.4. **(B)** Deviation of alpha carbons in repeats I-IV of Nav1.4 from the NavAb atoms, which are considered as matching according different sequence alignments. Introducing deletions decreases deviations and smoothes the deviation curves.

The P1 helices in the 3D aligned NavAb and Nav1.4 are similar and most deviations are less than 2 Å. Importantly, the four deviation curves in this region are smooth (**Figure 5D**). However, downstream the selectivity filter (position 50) the deviations sharply increase, resembling those between the 3D aligned Nav1.4 and KcsA. In this region, the deviation curves become highly irregular. Similar irregular curves are seen in the KcsA/Nav1.4 deviation plot at positions C-terminal to the selectivity filter where P-loops of these channels have different folding (**Figure 5C**). However, the irregular curves in the NavAb/Nav1.4 deviation plot are surprising because the folding of P1 and P2 helices in these channels is very similar. The cause of the irregular deviation curves is the straightforward sequence alignment downstream the selectivity filter. **Figure 5D** shows that residues in the matching position of P1 helices have similar 3D orientations, whereas orientations of residues in the P2 helices are dramatically different. To reconcile the sequence and 3D alignments, we introduced in the Nav1.4 sequence deletions (**Figure 6A**), which were proposed to build our model of TTX-bound Nav1.4 (Tikhonov and Zhorov, 2012). These adjustments removed the curve irregularity and sharply decreased deviations in repeats I, III, and IV (**Figure 6B**). However, in repeat II significant irregularity remained after position 54, indicating another problem in the alignment. Introducing the second deletion in repeat II of Nav1.4 solved the problem (green curve in **Figure 6B**).

**FIGURE 7:**
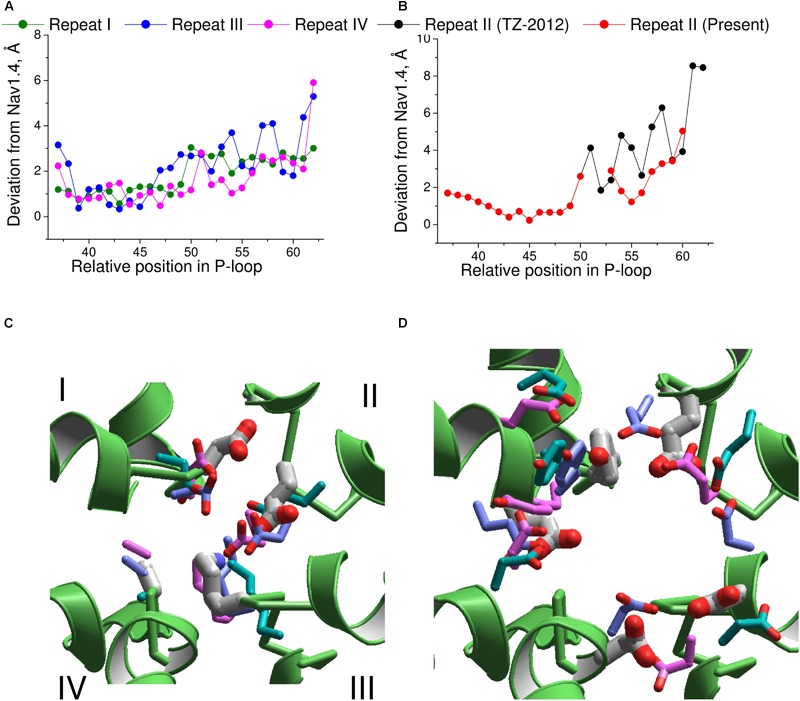
Comparison of the Nav1.4 cryo-EM structure with homology models. **(A)** P-loop alpha carbons in repeats I, III, and IV of the NavAb-based model (Tikhonov and Zhorov, 2012) are close to the matching atoms in the Nav1.4 structure. **(B)** In repeat II, big irregular deviations are seen because the Nav1.4 sequence aligned with NavAb lacks deletion at position 54 (**Figure 6A**). Introducing this deletion reduced deviations and the curve becomes similar to those in other repeats. **(C)** Side chains of the DEKA ring in 3D aligned structures. Only the Nav1.4 backbone is shown. The Nav1.4 residues are shown as thick sticks. Residues in the KcsA-based models (Lipkind and Fozzard, 2000; Tikhonov and Zhorov, 2005a) and NavAb-based model (Tikhonov and Zhorov, 2012) are shown, respectively, with cyan, magenta, and violet thin sticks. **(D)** Side chains of the outer carboxylates and a TTX-interacting tyrosine in position 51 of repeat I. Residues are rendered as in **(C)**.

Next, we compared the Nav1.4 structure with three homology models, which have been built to rationalize action of TTX and STX. Deviations from Nav1.4 are large (4–5 Å) in the model, which was built using the KcsA template modified to shift apart the P1 helices (Lipkind and Fozzard, 2000). Deviations of another KcsA-based model (Tikhonov and Zhorov, 2005a) from Nav1.4 are smaller, but they are still rather large (2–3 Å). The NavAb-based model (Tikhonov and Zhorov, 2012) is significantly more precise (**Figures 7A,B**). The largest deviations are seen C-terminal to position 54 of repeat II (**Figure 7B**), where necessity of the second deletion (**Figure 6**) was not recognized in lack of experimental data on TTX interactions with respective residues.

The above deviations of alpha carbons in the models from Nav1.4 obviously depend on the templates used for modeling. We further compared coordinates of the functional groups that interact with TTX, which served as a molecular probe to build the models. The general disposition of the TTX-interacting functional groups in all the models is similar to that in the experimental structure of Nav1.4 (**Figures 7C,D**). The outer carboxylates in the KcsA-based models (Lipkind and Fozzard, 2000; Tikhonov and Zhorov, 2005a) have rather large deviations from Nav1.4, whereas the NavAb-based model (Tikhonov and Zhorov, 2012) is much more precise (**Table 1**). The main outlier is the outer carboxylate in repeat III, which does not interact with TTX (Terlau et al., 1991) and therefore the models had been built without specific constraints between TTX and this carboxylate. Deviations of the functional groups in the NavAb-based model (Tikhonov and Zhorov, 2005a) are usually larger than respective deviations between NavPaS and Nav1.4. Of primary importance, however, is the fact that the deviations between two experimental structures of closely related eukaryotic sodium channels are in the same range as deviations of the NavAb-based model from the experimental structure (**Table 1**).

## Conclusion

Here we compared homology models Nav1.4, which are built using TTX, STX and μ-CTXs as molecular probes, with cryo-EM structures of eukaryotic sodium channels NavPaS and Nav1.4. Due to above-mentioned limitations of indirect approaches, including homology modeling, the exact predictions are hardly possible in lack of appropriate template structures. Nevertheless, several important structural features of sodium channel pore are predicted correctly. First, the predicted clockwise arrangement of the channel repeats at the extracellular view (Dudley et al., 2000) is now seen in NavPaS and Nav1.4. Second, the general configuration of the outer pore and mutual disposition of the selectivity filter residues and outer carboxylates in the KcsA-based models (Lipkind and Fozzard, 2000; Tikhonov and Zhorov, 2012) is correctly predicted despite the outer pore structure and the selectivity filter details in KcsA are dramatically different from those in Nav1.4. Third, docking of TTX into the NavAb-based model of the Nav1.4 channel allowed improving the sequence alignment between the prokaryotic and eukaryotic sodium channels. Fourth, experimental data on action of toxins helped to predict important features of sodium channels such as localization of the hydrophobic access pathway and presence of a hydrated sodium ion in the central cavity. Thus, experimental confirmation of predictions from structural studies of ion channels, in which toxins and other small molecules were used as molecular probes, justifies employment of this approach for future research.

## Author Contributions

All authors listed have made a substantial, direct and intellectual contribution to the work, and approved it for publication.

## Conflict of Interest Statement

The authors declare that the research was conducted in the absence of any commercial or financial relationships that could be construed as a potential conflict of interest.

## Abbreviations

μ-CTX: μ-conotoxins
EM: electron microscopy
LRET: luminescence resonance energy transfer
STX: saxitoxin
TTX: tetrodotoxin
VGSCs: voltage-gated sodium channels
VSD: voltage-sensing domain
